# A smartphone-based method for extraction-free antioxidant capacity analysis of emulsions on paper by the DPPH assay

**DOI:** 10.1016/j.mex.2025.103434

**Published:** 2025-06-19

**Authors:** Paraskevi-Evi Paraskevopoulou, Georgia Eleni Tsotsou, Foteini Chourda

**Affiliations:** a Laboratory of Chemistry, Biochemistry and Cosmetology, Department of Biomedical Sciences, University of West Attica, Egaleo, 122 43, Greece; b R&D Department, Anaveris SA, Ioannou Metaxa 56, Karellas, Koropi, 19400, Greece

**Keywords:** Digital image colorimetry, N-acetyl-l-cysteine, Resveratrol, Water-in-oil and oil-in water emulsion analysis, Smartphone camera, A smartphone-based method for extraction-free antioxidant capacity analysis of emulsions on paper by the DPPH assay

## Abstract

We are proposing an alternative quantitative analysis method for emulsions. The method relies on a simple and rapid protocol for antioxidant capacity measurement on chromatography paper by a digital smartphone camera and colorimetric analysis. It requires no extraction or pre-treatment step, as the emulsion is applied directly on the DPPH reagent spotted on a paper strip. Reliable calibration curves (R^2^>0.980) are built in an antioxidant-free base cream. Commercial samples are analyzed after significant dilution in the base cream, as antioxidant levels in cosmetics or many food emulsions are greatly above the linear dynamic range of the proposed format. The significant dilution zeroes out any matrix effect. Relative error ≤ ± 12.61 % was achieved upon analysis of commercial samples.

This method can be a greener and simpler tool, even in minimally equipped sites, for quality control of emulsions.

Method outline:

• Reflected light intensity measurements, by smartphone camera, can be an effective means for analysis of antioxidant capacity of emulsions.
• A simplified, extraction-free format on chromatography paper has been validated.
• The wide applicability of the paper-based format to the monitoring of antioxidant capacity of emulsions by smartphone, has been demonstrated for several antioxidants and different types of emulsion formulations.

## Graphical abstract

Illustration scheme for paper-based assay of antioxidant capacity directly in emulsions

**Figure:**
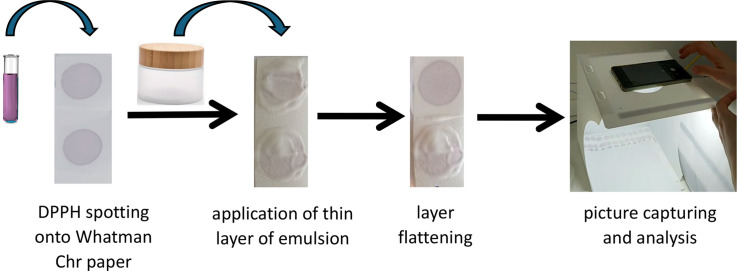

Specifications table

**Table:** 

Subject area:	Pharmacology, Toxicology and Pharmaceutical Science
More specific subject area:	*Emulsion analysis*
Name of your method:	A smartphone-based method for extraction-free antioxidant capacity analysis of emulsions on paper by the DPPH assay
Name and reference of original method:	Tsotsou, G.E., Paraskevopoulou, P.E., An extraction-free, smartphone-based approach for measuring the antioxidant capacity of emulsions using a paper-based DPPH assay, Microchem. J. 207 (2024), 111,792 https://doi.org/10.1016/j.microc.2024.111792
Resource availability:	

## Background

The photometric methods for antioxidant capacity measurement are typically run in solution [1], while paper-based formats have also been proposed, certain relying on the use of a smartphone camera [2,3]. In the specific case of emulsions, antioxidant capacity measurements require extraction of the analyte of interest prior to running the antioxidant assay in solution [4,5]. The reliability of this approach is questionable though, since antioxidant partitioning in emulsion, crucial for effective lipid protection, is not appropriately considered [1,[5], [6], [7], [8]]. Measurement of the protection offered by an antioxidant within an emulsion can only be achieved by running the DPPH reaction in the emulsion matrix. In oil-in-water (O/W) emulsions, the DPPH radical is diffused into the oil droplets, rendering its scavenging a measure of the actual protective effect of the studied molecule. In a previous publication we have demonstrated the suitability of a new analysis format for determining antioxidant capacity directly in O/W emulsions [9]. We have demonstrated in addition, that the proposed format was appropriate for studying the antioxidant capacity of little soluble compounds, like α-tocopheryl acetate. Such compounds cannot be studied by the conventional spectrophotometric assay in solution due to solubility restrictions [10]. The proposed setup provides a rather rapid and simple procedure which is more sustainable, with limited generated waste and lower cost than the conventional setup in solution after analyte extraction. It additionally offers easy digitalization of results, requires very little expertise and a minimal sample amount.

This method paper supplements the related publication [9] by providing a detailed description of the setup of the assay in paper format and of the effect of certain parameters on assay sensitivity and quantification accuracy. It additionally validates further the proposed format by studying the technical parameters upon analysis of additional antioxidants in O/W emulsions, namely N-acetyl-l-cysteine (NAC) and RESVERATROX® (INCI: Vitis Vinifera (Grape) Vine Extract (and) Butylene Glycol (and) Aqua). Finally, it demonstrates the assay's versatility by its suitability for monitoring the antioxidant potential of both water-in-oil (W/O) and O/W emulsions.

## Method details

1. Right before the start of the analysis, standards are prepared in a suitable emulsion matrix. An indicative O/W matrix composition is Aqua 81.7 % w/w, Paraffinum Liquidum 10 % w/w, Cetearyl Alcohol 4.0 % w/w, Glyceryl Stearate 3.0 % w/w, Phenoxyethanol 1.0 % w/w, Xantham Gum 0.2 % w/w, Ethylhexylglycerin 0.1 % w/w (emulsion base O/W). A typical suitable W/O matrix presents the following w/w composition: Aqua 70 %, Mineral Oil 15.0 %, Glycerine 5.0 %, Polyglyceryl-4 Diisostearate/Polyhydroxystearate/Sebacate 3.0 %, Microcrystalline Wax 3.0 %, Polyglyceryl-3 Diisostearate 2.0 %, Sodium Chloride 1.5 %, Phenoxyethanol 0.45 %, Ethylhexylglycerin 0.05 % (emulsion base W/O).
2. The unknown samples are diluted appropriately into the matrix of the standards to reach analyte concentrations within the linear dynamic range. Dilution factor should be of at least 100, to minimize any effect of the sample matrix. Several dilutions with different dilution factors might be required to ensure that an appropriate final concentration is reached. According to our experience, exact reproduction of the matrix between standards and unknowns is essential for minimizing matrix effect. For instance, if water is added in the standards to facilitate mixing, the same relative amount of water must be added in the unknowns to reproduce accurately the standard matrix.
3. DPPH Application: 10 µL of a DPPH solution (0.3–0.4 mg/mL) is spotted onto a strip of chromatography paper and is let to dry out. At this stage, it is recommended that the DPPH reagent volume is such that a free drop is released onto the paper strip. If the tip of the dispenser must touch the paper for the drop to be released, a non-uniform spread of the reagent might occur, that may subsequently deteriorate photo quality and results reproducibility.
4. Emulsion Application 1: A layer of approximately 1–2 mm of the emulsion (whether standard or sample) is applied on the spot. A plastic spatula rather than a metal one must be used, not to favorize oxidation reactions.
5. Emulsion Application 2: Within 10 s, a cover glass is pressed gently, but firmly, down onto the emulsion layer. This spreads the emulsion into a thin, even layer over the DPPH spot. The excess emulsion, that squeezes out from the edges, is scooped using the cover glass and discarded.
6. Picture Capturing: 5–20 min after emulsion application at room temperature, a picture of the spotted paper strips is captured by a smartphone camera. It is essential that the pictures are taken inside a white light box for accurate readings. The specific light box used, is a white photographic cube (23 cm x 23 cm), from Shenzhen PULUZ Technology Limited (China), equipped with a single row of light emitting diodes (LED). A 50-Megapixel mobile phone camera (parameters: Aperture f/1.9, Focal length 24 mm, 1x magnification factor) is used for picture capturing. The phone sits on top of the box, with the camera lens positioned over an opening in the top. To avoid glare, the paper strips are placed further away from the LED light strip.
7. Assay Setup: Typically, the assay is run in duplicate. Two spots are generated per paper strip, where the same standard, or sample is applied. The strips are then placed in the light box, side by side, with the spots positioned parallel to the LED light strip. For increased accuracy and reproducibility in analysis, ensuring the strips are perfectly flat is essential. If the number of samples is such that not all paper strips can fit in a single line, it is essential that a picture of each sample spot is captured in a photo that also includes the standards. In other words, it is crucial that the calibration curve used to quantify the unknown samples is built from standards included in the same picture. At least three pictures of each set of samples are taken within 20 min after emulsion application, with at least 5 min between pictures. The pictures are analyzed using the ImageJ software [11] as below. To ensure accuracy, the final concentration of the unknown samples is the average value calculated from pictures with calibration curves that have a strong correlation coefficient (R² ≥ 0.985).
8. Image Analysis: A selected area of the spot, typically around 3500–5000 square pixels is submitted to RGB image analysis by the image editing software ImageJ [11]. Any areas with non-homogenous coloration (e.g. at the edges of the spot) are not considered upon picture analysis. The values of the Red, Green, Blue channels are recorded and plotted against antioxidant concentration in the standards. The channel providing the best fit to the linear model (i.e. the green channel under the specific experimental conditions) is selected for generating the standard curve to calculate the unknown concentration.

### Further remarks

A. A variety of formulas are appropriate as emulsion matrix for the standards and diluent for the unknowns. We have successfully tested O/W formulas with viscosities varying from 420,000 cP to 1000 cP and pH values varying between 4.85 and 6.5.
B. Emulsion homogeneity is crucial for accurate measurements.
C. Thin-layer chromatography silica gel plates also do work as solid support for the spot test, providing a linear relationship between spot color intensity and antioxidant concentration in the applied emulsions. They are not, however, as easy to handle as chromatography paper and are thus more prone to contamination of neighboring spots and should not be preferred.
D. Colorimetric measurements performed by smartphone rely on the intensity of the reflected radiation. Although the intensity of a color is directly related to the amount of light it absorbs, it is inversely related to the light it reflects. For simplicity purposes, the analytical signal can be transformed after subtracting the color values (green component values in our case) from 255, the value corresponding to the total reflection of the radiation. In this way, the analytical response becomes proportional to the color intensity.
E. Based on the group’s experience, optimum monitoring channel might depend on cosmetic formula, DPPH concentration or even picture quality.
F. It is important that standards of antioxidants in the cosmetics matrix are prepared very soon before the analysis, to minimise antioxidant degradation.

## Experimental

### Apparatus and software

A Xiaomi Redmi Note 12 Pro 5 G smartphone with a 50-megapixel camera was used to capture images. A white photographic cube (23 cm edge) from Shenzhen PULUZ Technology Limited (China) equipped with LED lights was used for controlled lighting conditions. A Brookfield DV-E digital viscometer with Helipath T-bar spindles was used to measure emulsion viscosity at 20 °C. A Silverson L5M-A homogenizer was used for emulsion preparation. An InoLab pH Level 1 precision pH meter with a WTW SenTix 41 combination electrode was used for pH measurements. The ImageJ software [11] was employed to process the digital images.

### Materials and methods

N-acetyl-l-cysteine (NAC) 98.5–101.1 % w/w was provided by Cambridge Commodities. DL-α-tocopheryl acetate 96.5–102 % w/w was from BASF. RESVERATROX® (INCI: Vitis Vinifera (Grape) Vine Extract (and) Butylene Glycol (and) Aqua) was obtained from Actichem as a 2 % solution in a mixture of butylene glycol and water. 2,2-Diphenyl-1-(2,4,6-trinitrophenyl)hydrazyl (DPPH) was purchased from Sigma-Aldrich. NAC was prepared as a 0.3 % w/v solution in water, and RESVERATROX® solution was diluted with ethanol at a 1:1 ratio. A 0.3–0.4 mg/mL stock solution of DPPH in methanol was used in all experiments. Grade 1 Chr Cellulose Chromatography Paper from Whatman was used for the spot test. Different O/W or W/O emulsions, both in-house formulations and commercial products, were used as matrices for the assay. Their compositions and specifications are provided within the text.

### Method validation

A few studies from our group have validated the extraction-free, direct quantification of different analytes in O/W emulsions by a smartphone camera [12,13]. Regarding the assay on filter paper, initial method validation studies were published elsewhere [9]. These involved calculation of Half Maximal Effective Concentration (EC50) values towards common antioxidants and measurement of intra-assay precision and accuracy upon ascorbic acid quantification. Optimization of several factors (DPPH concentration, incubation time, color channel for analysis) was also described in the same work [9]. Further validation studies that highlight the width of the applicability of the proposed paper-based format are presented below:

**a) Calculation of the EC50 value of RESVERATROX®**

**Fig. 1:**
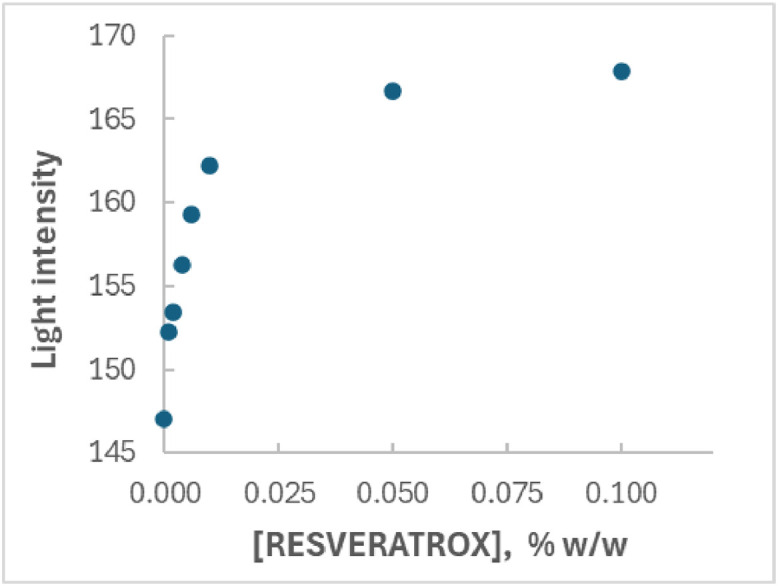
Typical dose-response curve depicting light intensity reflected by the spots on the paper strips versus RESVERATROX® concentration in an oil-in-water emulsion. The green color component was monitored of a digital image taken 25 min after emulsion application onto the dried DPPH spots. When fitting the data points to the 4PL function, a Pearson’s coefficient (R^2^) of 0.994 was obtained.

The commercially available antioxidant RESVERATROX®, offering antioxidant protection to keratinocytes [14,15], was incorporated in a O/W emulsion and was subjected to a dose/response study using the proposed assay format. The average EC50 value resulting from four dose/response curves was calculated to 0.00436 % w/w (i.e. 43.6 µg/mL considering the emulsion density of 1 g/mL, CV=44.7 %). RESVERATROX® is a French vine shoots extract containing resveratrol and its oligomers, well-known for their antioxidant properties. The calculated EC50 value compares well with literature values for resveratrol and its oligomers reported as 12 µg/mL for resveratrol [16], 36.1 µg/mL for pallidol [16], 34.9 µg/mL for cyphostemmin B [16], 94.6 µg/mL for trans-resveratrol [17] and 78.0 µg/mL for Scirpussin A [17]. A typical dose-response curve is given in Fig. 1.

**b) Analysis of commercial emulsions containing N-acetyl-l-cysteine**

We have previously demonstrated the linear relationship between NAC concentration in emulsions and antioxidant capacity, as assessed by the proposed method setup [9]. To further demonstrate the usefulness of the method in quantifying NAC in commercial formulations, mock formulations were prepared by adding NAC to an antioxidant-free, hand and body hydrating commercial cosmetic cream. NAC concentrations in the mock formulations were at levels comparable to those found in commercially available products. To ensure that upon antioxidant activity assaying, the antioxidant levels were within the assay's linear range, the mock formulations were diluted with a suitable emulsion, as matrix diluent. The diluent composition was Aqua 81.7 % w/w, Paraffinum Liquidum 10 % w/w, Cetearyl Alcohol 4.0 % w/w, Glyceryl Stearate 3.0 % w/w, Phenoxyethanol 1.0 % w/w, Xantham Gum 0.2 % w/w, Ethylhexylglycerin 0.1 % w/w (emulsion base O/W). In parallel, a set of standards (0 - 0.008 % w/w NAC) were built in the diluent matrix and a calibration curve was produced, applying the paper-based assay with the proposed protocol [9]. In all cases, standard curves with an R^2^>0.985, providing a high confidence level, were used to calculate NAC concentration in the mock formulations and the accuracy in terms of relative error (RE %). The resulting analysis parameters for the mock preparations are provided in Table 1.

**Table 1:** Accuracy of the proposed method for the quantitative determination of N-acetyl-l-cysteine in a commercial formulation.

[NAC] spiked, % w/w	Dilution in emulsion base O/W, prior to analysis	RE %	CV ( %) (n)
0.4	1: 80	+ 3.50	5.7 (*n* = 3)
0.65	1: 225	+ 5.64	33.7 (*n* = 5)
1.30	1: 484	+12.61	14.2 (*n* = 5)
7.40	1: 1945	- 4.16	29.1 (*n* = 5)

n: number of replicates.

RE: relative error.

CV: coefficient of variation.

**c) Analysis of the antioxidant potential of a W/O emulsion**

**Fig. 2:**
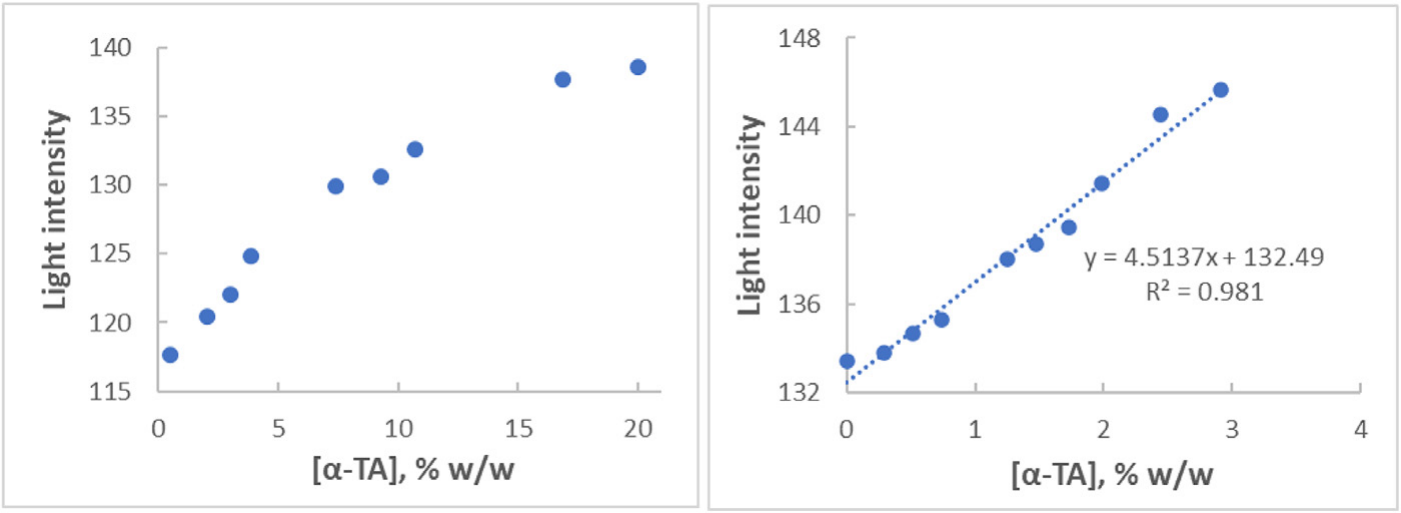
*Left:* Dose-response curve depicting reflected light intensity of the spots versus α-ΤΑ concentration in a water-in-oil emulsion. *Right*: Linear relationship between reflected light intensity of the spots on the paper strips and α-ΤΑ concentration in the same emulsion, at lower α-ΤΑ levels. The green color component was monitored using digital images taken 5–10 min after emulsion application onto the dried DPPH spots. A Pearson’s coefficient (R^2^) of 0.995 was obtained, when fitting the data points of the dose-response curve (left) to the 4PL function.

To investigate further the width of the applicability of the suggested protocol, a series of standards of α-tocopheryl acetate (α-TA), between 0 and 20 % w/w, were built in emulsion base W/O. Upon applying the paper-based DPPH assay, a linear relationship was observed between reflected light intensity and α-TA concentration for the lower concentration standards (Fig. 2, right), same as for an O/W emulsion containing α-TA [9]. For all standards employed in all six replicate linear calibration curves (R^2^≥0.981), the standard concentration was back-calculated within ± 16.6 % of their nominal concentration (± 42.7 % at Lower Limit of Quantification (LLOQ)), indicating acceptable accuracy. Moreover, dose-response curves were built (Fig. 2, left) which displayed a good fit to the 4 parameter logistic (4PL) function (R^2^≥0.985). Using four replicate dose-response curves, the average EC50 value was calculated to 23.64 % w/w (CV=66.2 %). This value is about 3 times higher than the value calculated for α-TA in an O/W emulsion by the same assay format [9] It is not clear at this stage, if the observed difference is related to the structural characteristics of the different types of emulsions.

Concluding, this study successfully expanded upon previously established validation of the paper-based DPPH assay, demonstrating its versatility and applicability to a wider range of antioxidant compounds, and different types of emulsion formulations. The consistent to the literature determination of EC50 value for RESVERATROX®, a complex plant extract, together with the accurate quantification of N-acetyl-l-cysteine in a commercial emulsion, and preliminary validation results in a W/O emulsion, collectively solidify the potential of applying this assay as a valuable tool in various research and industrial settings.

Unlike typical methods for testing the antioxidant capacity of emulsions (e.g. in [4,5]), the proposed method eliminates the need for prior extraction of the antioxidant(s). This is a significant improvement, as extraction can compromise the reliability of the measurement by not accurately reflecting the antioxidants’ partitioning within the emulsion [1,[5], [6], [7], [8]]. While other paper-based formats using smartphone cameras exist for antioxidant activity analysis after extraction [2,3], this method specifically adapts the DPPH assay for direct use within emulsions, addressing the unique challenges posed by emulsion matrices. An additional advantage is that being solvent-free, with minimal reagent and sample consumption, it significantly reduces environmental impact and disposal requirements. Moreover, the reduced chemical usage and lack of liquid waste lead to lower costs and diminish health risks associated with solvent exposure.

## Limitations

The relevance of the antioxidant capacity-measuring colorimetric assays, digital or not, to the actual capacity of the antioxidant molecule to scavenge short-leaving free radicals within real emulsions has been questioned in the literature [18]. Although this weakness is valid for the proposed setup, our modifications to the conventional assay do take at least into consideration antioxidant partitioning in emulsion. Antioxidant partitioning is a crucial parameter determining the efficacy of lipid protection [[5], [6], [7], [8]].

The proposed assay is applied directly in emulsions and provides sufficient accuracy in those samples where antioxidant levels are sufficiently high (at least 100-fold higher than the upper limit of the linear dynamic range). According to our experience, the effect of the matrix itself on the reflected light captured by a digital camera is significant and matrix dependent [9]. This effect cannot be minimized in the absence of significant dilution of the sample matrix in the diluent matrix. In the absence of appropriate dilution, accuracy of determination deteriorates. However, in most applications in the cosmetics industry and in several applications in the food industry, antioxidant levels are much higher than 100 times the LOQ of the proposed method, as determined by our group [9]. For samples with lower antioxidant content, the sample matrix (free of antioxidant) should become available to the analyst, to be used as blank. It is, hence, crucial that the analyst evaluates sample characteristics in relation to the assay's limitations to ensure valid data interpretation.

## Ethics statements

No ethics statements are applicable.

## CRediT author statement

**Paraskevi-Evi Paraskevopoulou:** Investigation, Formal analysis, Writing – review & editing; **Georgia Eleni Tsotsou:** Conceptualization, Methodology, Validation, Investigation, Formal Analysis, Writing - original draft, Writing – review & editing; **Foteini Chourda:** Investigation, Formal analysis.

## Declaration of competing interest

The authors declare that they have no known competing financial interests or personal relationships that could have appeared to influence the work reported in this paper.

## Data Availability

No data was used for the research described in the article.

## Appendix Supplementary materials

Supplementary material *and/or* additional information [OPTIONAL] *Video depicting the steps of the proposed assay format* During the preparation of this work, the authors used Gemini in order to improve language and readability. After using this tool/service, the authors reviewed and edited the content as needed and take full responsibility for the content of the publication. Image, video 1
